# Mindfulness‐Based Orofacial Pain Education and Management Program: A Qualitative Analysis of Transgender Participants’ Perceptions

**DOI:** 10.1111/scd.70141

**Published:** 2026-01-28

**Authors:** Melissa de Oliveira Melchior, Beatriz Dorne, Alex Moreira Mélo, Nadyne Saab Messias, Victor Hugo Alves Ribeiro‐Silva, Lucas Gaspar Ribeiro, Lúcia Alves da Silva‐Lara, Christie Ramos Andrade Leite‐Panissi, Edilaine Cristina da Silva Gherardi‐Donato, Jardel Francisco Mazzi‐Chaves, Laís Valencise Magri

**Affiliations:** ^1^ Departamento De Odontologia Restauradora Faculdade De Odontologia de Ribeirão Preto Universidade De São Paulo Ribeirão Preto São Paulo Brazil; ^2^ Departamento De Psicologia Faculdade De Filosofia Ciências e Letras de Ribeirão Preto Universidade De São Paulo Ribeirão Preto São Paulo Brazil; ^3^ Departamento De Clínica Médica Faculdade De Medicina de Ribeirão Preto Universidade De São Paulo Ribeirão Preto São Paulo Brazil; ^4^ Departamento De Ginecologia e Obstetrícia, Faculdade de Medicina de Ribeirão Preto Universidade De São Paulo Ribeirão Preto São Paulo Brazil; ^5^ Departamento De Enfermagem Psiquiátrica, Escola de Enfermagem de Ribeirão Preto Universidade De São Paulo, PAHO/WHO Collaborating Centre For Nursing Research Development Ribeirão Preto São Paulo Brazil

**Keywords:** mindfulness, orofacial pain, pain education, temporomandibular disorders, transgender health

## Abstract

**Introduction:**

Transgender individuals are disproportionately affected by temporomandibular disorders (TMDs), often compounded by psychosocial stressors and limited access to affirming healthcare. Conventional TMD treatments may fall short in addressing the emotional and behavioral components of chronic orofacial pain, underscoring the need for integrative interventions. This study aimed to explore the implementation and perceived impact of a Mindfulness‐Based Orofacial Pain Education Program as a complementary approach for transgender individuals with TMD.

**Methods:**

A qualitative design was employed, using weekly instructor reports and a post‐intervention focus group with transgender participants (ages 24–39), each diagnosed with TMD according to the DC/TMD criteria. Over eight weeks, participants attended weekly 60‐min sessions incorporating mindfulness practices, pain education, and self‐management strategies. Data sources included 8 weekly instructor audio reports (*n* = 32 recordings) and one focus group interview (duration: 68 min). All transcriptions were analyzed using Thematic Network Analysis supported by NVivo software (QSR International).

**Results:**

The Analysis identified a global theme of increased self‐awareness and engagement in pain management. Three organizing themes emerged: (1) social support as a catalyst for adherence; (2) mindfulness as a strategy for emotional and sensory regulation; and (3) environmental and motivational barriers to sustained practice. Participants reported qualitative improvements in emotional regulation, sleep quality, and pain perception. Challenges included distraction due to the shared clinic setting and difficulties maintaining routine mindfulness practice, especially among participants reporting lower initial motivation.

**Conclusions:**

The Mindfulness‐Based Orofacial Pain Education Program was perceived as a feasible and beneficial adjunct to standard TMD care, promoting self‐efficacy, emotional resilience, and pain self‐management in a socially vulnerable population. Despite the small sample, consistent thematic convergence and participant engagement suggest promising applicability.

## 1 Introduction

Orofacial pain, particularly that associated with temporomandibular disorder (TMD), is a prevalent condition that significantly impacts individuals’ quality of life. Patients with orofacial pain often experience functional limitations, chronic pain, and psychological effects such as anxiety and depression [1, 2, 3]. Mood disturbances such as anxiety and depression are known to exacerbate pain perception and contribute to its chronicity by amplifying central sensitization and stress‐related physiological responses [1, 3, 4]. In TMD specifically, higher anxiety and depressive symptom scores are consistently associated with greater pain intensity, muscle tension, and impaired coping ability [2, 4, 5]. Conventional approaches such as occlusal splints, physiotherapy, and pharmacological management have demonstrated efficacy in reducing pain intensity and restoring masticatory function for a substantial proportion of TMD patients. However, their long‐term effectiveness remains limited because these interventions primarily target physical symptoms and neglect psychological and behavioral dimensions that contribute to pain persistence, maladaptive coping, and treatment dropout. Therefore, complementary strategies that integrate emotional regulation and self‐management—such as mindfulness‐based education—are warranted to achieve more sustainable outcomes [4, 5, 6].

Transgender individuals face significant social vulnerability, which affects their overall health and access to care. Stigmatization, discrimination, and lack of professional training in transgender health contribute to the limited access to health services for this population [7, 8, 9]. Social exclusion and minority stress increase the risk of emotional disorders such as anxiety and depression, which are directly associated with chronic pain conditions [10]. Additionally, harmful coping mechanisms such as tobacco and alcohol use are more prevalent among transgender individuals, further exacerbating their vulnerability to health problems. These combined factors emphasize the importance of adopting a biopsychosocial approach to address the multifactorial nature of orofacial pain in transgender populations [11, 12]. Emerging evidence suggests that transgender and gender‐diverse individuals may experience higher prevalence of DTM and related orofacial pain, likely due to chronic stress exposure, hormonal influences, and barriers to accessing affirming health care [10, 11, 12]. A study published in 2025 reported a significantly higher prevalence of TMD among transgender men compared to transgender women, with strong associations between TMD, anxiety, and alcohol consumption [10].

Thus, the search for complementary interventions that can provide more holistic and sustainable relief for these patients is justified. In this context, mindfulness has proven to be a promising tool, as it promotes present‐moment awareness, reduces stress, and enhances the response to pain treatment [13, 14]. Mindfulness refers to the ability to sustain moment‐to‐moment attention in an open, curious, and compassionate manner, allowing individuals to notice the variations that occur throughout their own experiences, including pleasant, unpleasant, or neutral events. It is an innate trait of personality but can also be cultivated through attentional practices in daily activities and meditation [15, 16].

Studies suggest that mindfulness can enhance emotional regulation and pain perception through cognitive modulation involving higher‐order brain circuits, thereby contributing to improved treatment adherence and patient self‐efficacy [16, 17, 18, 19]. By integrating mindfulness practices with education on TMD, the aim is not only to reduce pain and improve physical functionality but also to enhance patient satisfaction with treatment and improve overall quality of life. Thus, the objective of this study was to implement and evaluate a Mindfulness‐Based Orofacial Pain Education Program for transgender individuals receiving treatment at a specialized TMD and orofacial pain public service.

## 2 Methods

### 2.1 Study Design

This study employed a qualitative design, drawing from weekly instructor reports and a post‐intervention focus group. Data sources included weekly instructor reports and a post‐intervention focus group, enabling a comprehensive understanding of participants’ engagement and perceived impact throughout the program [20]. The research was conducted at the TMD and Orofacial Pain Clinic of the XXXXXXXXXXXXXXXXX. The primary objective was to explore the implementation and impact of a Mindfulness‐Based Orofacial Pain Education Program as a complementary intervention to conventional treatment.

In addition to participants’ narratives, facilitators’ weekly reports were included as a secondary qualitative data source. Facilitators provided an external, process‐oriented perspective on participants’ engagement, group interaction, and behavioral changes observed throughout the sessions. This approach allowed methodological triangulation, capturing both experiential and observational dimensions of the intervention.

Reporting adhered to the Consolidated Criteria for Reporting Qualitative Research (COREQ) guidelines. The completed COREQ checklist is provided as Supplementary Material 1.

### 2.2 Ethical Considerations

The study protocol was approved by the Research Ethics Committee (CAAE: XXXXXXXXXXXXXX), in accordance with the guidelines of Resolution No. 466/2012 of the Brazilian National Health Council. All participants provided written informed consent before enrollment, ensuring voluntary participation and data confidentiality. Ethical principles, including autonomy, beneficence, and non‐maleficence, were strictly followed throughout the study.

### 2.3 Participants and Selection Criteria

Participants were recruited using a convenience sampling approach based on the admission flow at the TMD/OFP clinic. Individuals aged 18 years or older, with a clinical diagnosis of TMD confirmed using the Diagnostic Criteria for TMDs (DC/TMD) [21] and receiving regular treatment at the specialized clinic were eligible to participate. Additionally, participants were required to have sufficient cognitive and linguistic ability to comprehend instructions and express themselves verbally. All patients present in the waiting room during clinic hours were invited to participate in the study, with the number of individuals varying according to patient flow.

A total of fifteen individuals completed the Mindfulness‐Based Orofacial Pain Education Program, which was offered to all patients attending the TMD and Orofacial Pain Clinic waiting area during the intervention period. At the conclusion of the program, all participants were invited to take part in the qualitative phase of the study. Four transgender participants (ages 24–39 years; two trans women and two trans men) consented to participate in the focus group. The remaining eleven participants declined participation primarily due to scheduling constraints or discomfort with audio recording. Although small in number, this sample is consistent with qualitative research principles, allowing in‐depth exploration of experiences within a socially vulnerable population.

Recruitment followed a convenience sampling strategy based on the regular flow of patients attending the clinic. Exclusion criteria encompassed individuals with neurological conditions that affected communication or cognitive function, prior structured experience with mindfulness‐based interventions, and acute psychiatric instability; no individuals met criteria for exclusion. Participants were also stratified by TMD severity using standardized diagnostic criteria. Demographic information is summarized in Table 1.

**TABLE 1:** Demographic and clinical characteristics of participants enrolled in the Mindfulness‐Based Orofacial Pain Education Program.

Participant	Age (years)	Gender identity	TMD diagnosis	Symptom duration	Employment/Education status
P1	24	Trans woman	Myofascial pain	6 months	Undergraduate student
P2	27	Trans man	Myofascial pain	1 year	Freelancer
P3	32	Trans woman	Myofascial pain	3 years	Hairdresser
P4	39	Trans man	Myofascial pain	5 years	University staff

*Note*: Data represent self‐reported demographic and clinical characteristics of the four participants who completed the Mindfulness‐Based Orofacial Pain Education Program and participated in the focus group. All participants met the *Diagnostic Criteria for TMDs* (DC/TMD) for myofascial pain. Symptom duration refers to the time since the onset of chronic orofacial pain as reported by each participant.

### 2.4 Mindfulness‐Based Orofacial Pain Education Program

The Mindfulness‐Based Orofacial Pain Education Program was developed by a multidisciplinary team of specialists in restorative dentistry, psychology, and nursing, all with expertise in orofacial pain and mindfulness‐based interventions. The program was designed as a structured, integrative educational protocol addressing both physical and psychosocial dimensions of TMD‐related pain. Its conceptual foundation combined three evidence‐based frameworks: Mindfulness‐Based Stress Reduction (MBSR), emphasizing attentional regulation, nonjudgmental awareness, and acceptance; the Explain Pain educational model, promoting understanding of pain neurophysiology and cognitive reframing of pain as a protective process; and the biopsychosocial model of TMD management, integrating biological, emotional, and social determinants of pain and treatment adherence.

A preliminary pilot phase with volunteer patients (*n* = 5) allowed refinement of session pacing, language accessibility, and group activities before implementation. The final structure incorporated three core components each week: (1) pain education addressing neurophysiological mechanisms, psychosocial influences, and self‐care strategies; (2) mindfulness‐based practices such as guided breathing, body scan, and awareness of jaw tension; and (3) self‐management exercises involving gentle orofacial and cervical movements to reduce muscle tension and enhance proprioceptive awareness. The program was delivered over eight weekly 60‐min sessions, with four to nine participants per session and two to four trained instructors certified in mindfulness‐based interventions and experienced in orofacial pain management.

At the initial consultation, participants received an educational booklet covering TMD mechanisms, psychosocial contributors, and self‐care recommendations; this material was revisited throughout the program to support engagement and knowledge retention. Each session followed a structured sequence: review of previous content and participant reflections, guided mindfulness practice, group discussion on challenges and benefits of the exercises, and closing reflections with suggestions for home practice. Mindfulness techniques included body scanning to increase sensory awareness, focused breathing to promote relaxation, and jaw movement monitoring to identify habitual tension patterns. Participants were encouraged to integrate these strategies into daily routines to foster self‐regulation of pain and stress. The post‐intervention focus group was conducted by an external researcher who were not involved in the sessions, minimizing interviewer bias.

### 2.5 Qualitative Analysis

The focus group guide was collaboratively developed by the multidisciplinary research team, informed by previous literature on mindfulness‐based interventions for chronic pain and TMD. Its content validity and clarity were reviewed by two external experts, pilot tested with two volunteers and refined before use. The semi‐structured guide included open‐ended questions exploring participants’ experiences with mindfulness practices, perceived benefits and challenges, barriers to engagement, social support influences, and suggestions for improvement.

**FIGURE 1:**
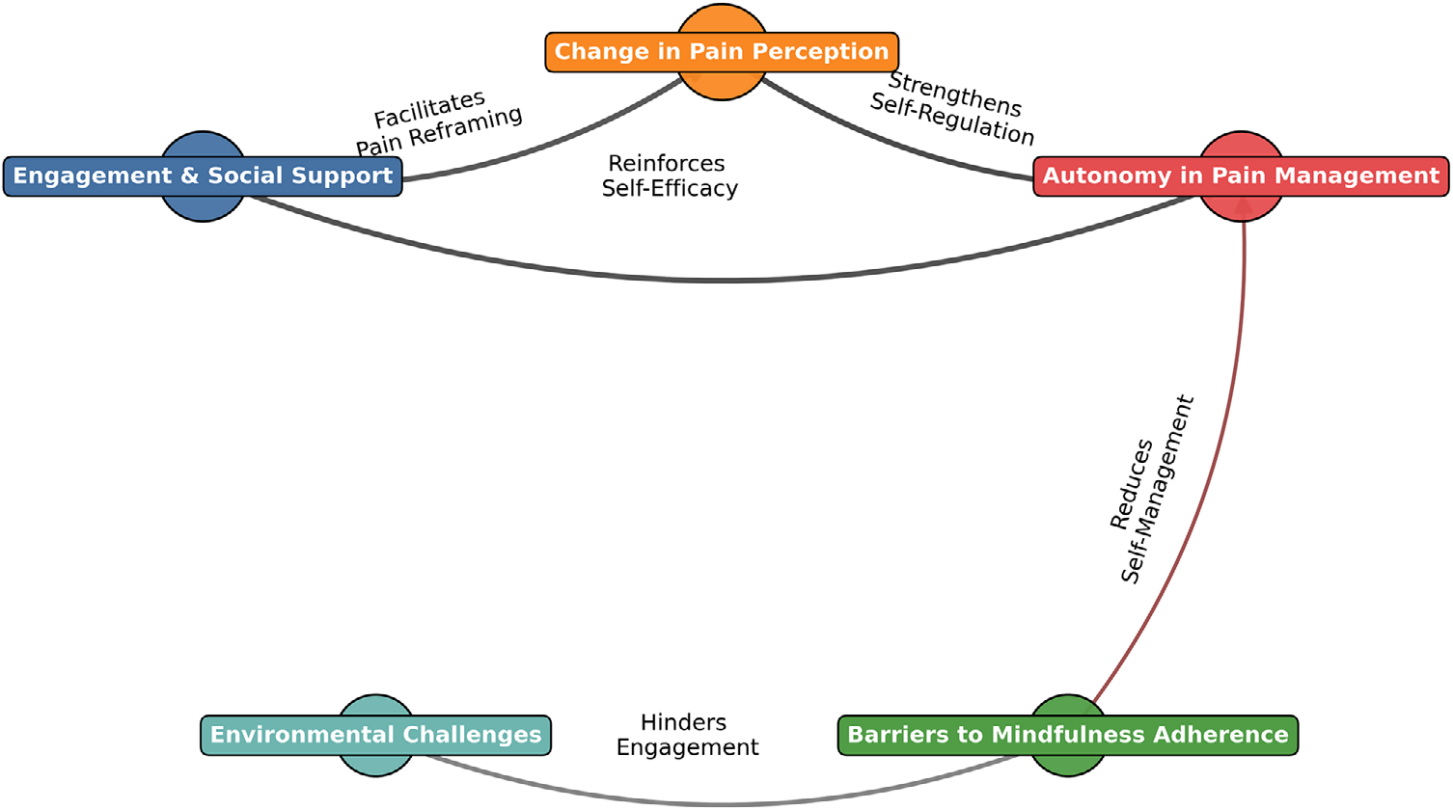
Thematic network of mindfulness‐based orofacial pain management. The figure visually summarizes the relationships among themes identified through thematic network analysis, illustrating how social support, pain reframing, autonomy in pain management, and contextual barriers were interconnected in participants’ and facilitators’ accounts.

All instructor audio reports and the focus group interview were transcribed using Google Docs and reviewed for accuracy. Qualitative data were analyzed using Thematic Network Analysis to identify global, organizing, and basic themes, supported by NVivo software for coding and data organization. The analysis explored how participants described their engagement and perceived benefits. Facilitator and participant perspectives were compared for triangulation; themes were mapped (Figure 1) and summarized with illustrative quotes (Table 2). To ensure trustworthiness, two researchers independently coded the data and reconciled discrepancies through discussion.

**TABLE 2:** Comparative analysis of perceptions: Instructors’ and participants’ perspectives on a mindfulness‐based Orofacial Pain Education Program.

Themes	Instructors’ perspective	Participants’ perspective
Welcoming and engagement	Participants exhibited initial receptiveness; however, the clinical environment‐imposed constraints on focus and participation.	The group dynamic facilitated identification with peers, fostering a sense of belonging.
Pain perception	Enhanced comprehension of the interplay between emotional states and pain perception.	Previously lacked awareness of pain etiology; participation contributed to recognizing triggers and modulating responses.
Mindfulness and self‐regulation	Some participants encountered challenges in sustaining mindfulness practices consistently.	Mindfulness techniques were perceived as beneficial, although certain individuals reported monotony in repetitive exercises.
Program impact	Participants demonstrated increased autonomy in pain management strategies, integrating learned techniques into daily life.	Improved self‐efficacy, enhanced psychological well‐being, and perceived gains in overall quality of life.
Recommendations for program enhancement	Identified need for diversification of intervention strategies to enhance engagement and effectiveness.	Preference for a more structured and private environment to minimize external distractions and optimize participation.

*Note*: The analysis was conducted using Thematic Network Analysis [22] to map emerging themes and explore interconnections between participant and instructor perspectives.

### 2.6 Researcher Positionality and Reflexivity

The research team comprised female investigators with professional backgrounds in dentistry, psychology, and mindfulness interventions, all with experience in orofacial pain management and mindfulness‐based interventions. The authors acknowledge that their clinical and educational experience may have influenced the interpretation of participants’ narratives. To enhance reflexivity and minimize bias, team discussions were conducted throughout data analysis to examine assumptions, challenge interpretations, and reach consensus on emergent themes.

## 3 Results

The analysis explored participants’ and facilitators’ perceptions of the Mindfulness‐Based Orofacial Pain Education Program, revealing that mindfulness practices fostered self‐awareness, emotional regulation, and greater autonomy in pain management. Group interaction strengthened social support, while environmental and motivational barriers influenced engagement. These findings address the study aim of exploring participants’ experiences with mindfulness‐based orofacial pain education. As summarized in Table 2, comparative analysis and Thematic Network Analysis provided a multidimensional understanding of participants’ and facilitators’ experiences.

The thematic network analysis identified a dynamic interplay between engagement and social support, changes in pain perception, autonomy in pain management, environmental challenges, and barriers to mindfulness adherence (Figure 1). Participants described that group interaction supported pain reframing from a biomedical to a biopsychosocial perspective, facilitating greater autonomy in pain management. This cognitive reframing was a key determinant in fostering autonomy in pain management, enabling participants to implement self‐regulation strategies more effectively. However, environmental challenges, such as external distractions and lack of privacy, emerged as significant barriers to mindfulness adherence, further compounded by internal obstacles, including perceived repetitiveness and motivation loss. Environmental factors, such as external noise and limited privacy, were reported as barriers to sustained engagement.

The thematic analysis identified four overarching themes that characterized participants’ and instructors’ experiences in the mindfulness‐based Orofacial Pain Education Program. First, cultivating mindful awareness, self‐compassion, and emotional regulation emerged as central, with participants reporting increased sensory awareness and a more accepting relationship with their bodies. Second, the power of group dynamics and shared experience was evident, as participants highlighted the value of social support and validation within the group setting. Third, understanding pain mechanisms and empowering self‐management was facilitated through pain education and practical techniques for coping with orofacial pain. Finally, navigating contextual barriers and personalizing engagement underscored the importance of addressing individual challenges and tailoring program elements to meet diverse needs and circumstances (Table 3, ANNEX 1).

**TABLE 3:** Thematic analysis of participants and instructors’ experiences: Themes and sub‐themes.

Theme	Sub‐themes	Illustrative quotes
**1. Cultivating mindful awareness, self‐compassion, & emotional regulation**	Enhancing sensory awareness: Increased awareness of muscle tension in the face, jaw, and neck; improved perception of jaw movement, breathing patterns, posture, and general comfort. Mindfulness for emotional regulation & pain management: Utilizing mindfulness practices to regulate emotional responses to pain (e.g., stress, anxiety), manage pain sensations, and cultivate a sense of acceptance and non‐judgment. Fostering self‐compassion and acceptance: Encouraging a gentle, kind, and accepting attitude toward one's own body, pain, and emotions, reducing self‐criticism and promoting self‐care and self‐soothing behaviors.	“They were able to feel it, right? We invited everyone to put their hands on their face, to better perceive the contraction when they clench their teeth…” “Bringing attention to the breath, attention to the body, attention to pleasant and unpleasant physical sensations…” “Pay attention to your own body, find a comfortable position, observe your breathing…” “Inviting to feel better in the body.” “We did another mindfulness practice, bringing attention to the breath, attention to the body, attention to pleasant and unpleasant physical sensations, and seeking to welcome all sensations…” “Bringing attention to the breath, without causing interference, without forcing breaths.” “Bringing the knowledge of what is necessary for me to feel better, right?” “But rather that they be reflections in a gentle way with oneself.” “We also talked about factors that influence the triggering of this alarm… The instructor gave some examples that for her, poor sleep greatly influences it…” ‘’To notice these symptoms with full attention, in an atmosphere of gentleness, welcoming with one's own body, of curiosity…‘’ “it's important to develop calmness”
**2. The healing power of group connection & shared experience**	Sharing personal pain narratives: Openly sharing individual experiences with orofacial pain, treatment journeys, coping strategies, and related personal challenges (e.g., sleep disturbances, stress). Creating a supportive community: Establishing a sense of community, reducing feelings of isolation, and providing a safe space for vulnerability, mutual understanding, and reciprocal emotional support among participants. Empowerment through shared learning: Facilitating the exchange of insights, knowledge, and practical strategies through peer‐to‐peer learning and mutual encouragement, fostering a sense of empowerment and collective problem‐solving.	“She said the same thing. She reported that she has sleepwalking, so when she rests more, the sleepwalking happens less…” “The participants shared personal experiences that altered their pain sensations.” “A participant also opened up a lot and she reported this issue that she sometimes feels very alone and that these conversation groups help her a lot.” “The participants reported that they greatly appreciate the activities carried out, highlighting that the conversation groups have contributed both to dealing better with their pains…” “He directed the guidelines so that they would pay attention, once again, to the experiences they were having during the week and that they could also share…because this helps each other, right?”
**3. Understanding pain & empowering self‐management strategies**	Pain education (explain pain framework): Providing education about pain mechanisms, distinguishing between acute and chronic pain, explaining the “alarm system,” identifying contributing factors, and emphasizing the difference between pain and tissue damage. Practical self‐management techniques: Implementing practical strategies for alleviating pain and enhancing overall well‐being, including jaw exercises, relaxation techniques, heat/cold therapy, and addressing lifestyle factors like sleep, diet, and stress management. Focus on tongue positioning & jaw stability: Highlighting the importance of proper tongue positioning for achieving jaw stability and reducing muscle tension in the face, jaw, and neck.	“We got into this talk about chronic pain and acute pain, about the difference, about how acute pain we go, we we treat, we change our whole lives to be able to treat that pain, but chronic pain we end up letting it accompany us during our lives…” “If I can't stop eating because I get home hungry, then can I eat something a little lighter? or can I take care of my sleep hygiene by drinking tea before bed?” “Next, the participants performed movements aimed at lubricating the joints…” “I told them, I said I was going to try to schedule a speech therapy evaluation… We talked about the positioning of the tongue…”
**4. Navigating barriers and facilitators to mindfulness engagement**	Reducing environmental influences: Recognizing and mitigating environmental factors (e.g., noise, distractions) that might negatively impact participants’ comfort, focus, and full engagement in the program. Optimizing group dynamics & inclusion: Skillfully managing group dynamics (e.g., new vs. returning members), building trust, and ensuring that all participants feel included, valued, and comfortable sharing their experiences. Tailoring to individual needs & circumstances: Acknowledging and accommodating individual needs, physical limitations, and practical constraints to ensure that strategies are tailored to each participant's unique situation and preferences.	“Maybe the environment of the clinic can also influence how comfortable they feel, because there are always people passing behind us, right?” “Maybe the fact that there is movement of new and old people, and the group is constantly mixing, maybe that intimidates some participants at times, right, from exposing themselves.” “Ah, that's impossible for me, I can't, I work, I study late, I get home hungry, I can't take care of my sleep hygiene…”

**ANNEX 1:** Mindfulness‐based orofacial pain education.

Week	Topic	Educational approach	Activities
**Week 1**	**Equalizing Expectations: What Can You Do for Yourself?**	Provides an overview of the content that will be covered throughout the program. Participants have an initial opportunity to express how they feel and what they expect from the program. Through guidance from the instructors, they begin to recognize their internal tools to face pain, such as a breathing body and a mindful mind. They learn that mindfulness is an innate and natural practice that brings personal benefits and enhances readiness for learning about their clinical condition. Participants start to understand that knowledge about Temporomandibular Disorders (TMD) and Orofacial Pain (OFP) will add valuable resources for managing their condition.	Group dynamic to foster connection: Each participant writes their expectations on post‐its, forming a mural that serves as a starting point for reflections and discussions about individual purposes and program objectives. This allows alignment of expectations and informed decision‐making about participation. Presentation of the Orofacial Pain Education Program incorporating mindfulness strategies, including duration, purpose, and types of activities.
**Week 2**	**Developing Calm, Enhancing Attention**	Breathing serves as the anchor of the mindfulness experience. This session begins by fostering calmness and physical comfort to enhance attention, learning, and self‐awareness in the present moment. Participants explore the rhythm, depth, and natural flow of their breath, developing a sensory perception of the body rather than cognitive assumptions. The discussion introduces fundamental structures involved in TMD, such as the temporomandibular joint (TMJ) and masticatory muscles. With mindful awareness, participants observe their jaw movements and related sensations with curiosity, openness, and kindness. To support the program, an educational leaflet is provided, containing information on pain, TMD, and management strategies.	Mindfulness practice focusing on body and breath awareness with openness and curiosity, free from judgment. Definitions of TMD, TMJ, and masticatory muscles and their functions. Group interaction: Noticing sensations in muscles and TMJ movements. Group reflections and discussions guided by instructors.
**Week 3**	**Awareness of “Pain” and “No Pain” in the Body**	Participants are encouraged to recognize pain as one of the many sensations in the present moment. A gentle, curious, and welcoming attitude toward the body—including pain—is cultivated to support proactive responses rather than reactive behaviors that may amplify suffering. Discussions explore how thoughts, emotions, and situations influence pain perception. The body serves as a tool for mindfulness practice, restoring the mind‐body connection as an inseparable whole. By shifting focus, participants acknowledge the presence of neutral and pleasant sensations often overshadowed by pain. The session introduces the physiological and dysfunctional pain systems, integrating participants’ innate knowledge. Understanding factors that contribute to pain and beneficial attitudes promotes confidence and assertiveness in managing their condition.	Body awareness mindfulness practice, emphasizing the orofacial region. Reflection and sharing about personal experiences throughout the week. Education on physiological and dysregulated pain and its management. Invitation for self‐observation: Noting what improves or worsens pain. Self‐care guidance: Applying moist heat (20–30 min) and deep muscle massage (1 min) daily.
**Week 4**	**Releasing and reducing tension**	With openness and curiosity toward the complete experience of the present moment, this week addresses the possibility of releasing and “letting go” with each breath, reducing orofacial and bodily tensions. Participants are encouraged to perceive the difference between tense and relaxed states through guided exercises involving intentional tension and breath‐holding, noticing the body's wholeness. They then freely release their breath and tension, observing how the body feels when letting go without restriction, keeping the jaw relaxed so that air can exit through the mouth and the orofacial muscles can loosen. Mindful awareness of these possibilities fosters acceptance of the current state and reduces self‐criticism, anxiety, and associated stress. These experiences open the door to discussions about sleep and awake bruxism, adding valuable insights into self‐care strategies and management techniques for different manifestations of bruxism, which may contribute to the pain experience.	Brief mindfulness practice focused on detachment and releasing bodily tensions. Activity on sleep and awake bruxism: Explanations and group reflections. Guided exercise on tensing and relaxing different body parts, including the masticatory muscles. Insights and discussions on stress and anxiety in modern life. Self‐care strategies and management techniques for different manifestations of bruxism, anxiety, and stress. Group training: Breathing out through the mouth while relaxing the jaw. Massaging the masticatory and cervical muscles. Applying warm compresses (15–30 min) to tense or painful muscles. Guidance on habits that overload the masticatory muscles (e.g., nail‐biting, chewing gum, etc.). Weekly suggestions: Finding personal ways to monitor orofacial and cervical tensions: Post‐it notes, apps, chamomile tea, gentle bedtime preparation, etc. b. Inviting oneself to practice the activities introduced in the session throughout the week.
**Weeks 5 & 6**	**“Moment by Moment: Facing Challenges”**	Gradually, gentle strategies for habit changes and self‐management are incorporated to deal with everyday challenges through mindfulness. The approach is designed to develop awareness of the impermanence of physical and mental states, noticing that changes constantly occur in the body, in breathing, in appearance, in the environment, and in others. This awareness includes understanding that external and internal events within the body happen beyond our control, but we can control how we respond to these changes. This is done by cultivating a gentle, curious, and non‐reactive perspective on various aspects of life, including nutrition, sleep, physical and orofacial exercises, and harmful oral habits, all aimed at promoting overall well‐being. This gradual recognition of transience fosters the concept of detachment. At this stage, guidance on dietary regulation, sleep hygiene, and physical exercise—along with orofacial self‐management exercises for TMD—is discussed. Insights into current habits serve as a basis for reflection on new possibilities within an individual context, respecting each participant's pace and the necessary time to ensure adherence and effective goal achievement. Small changes with self‐care and self‐compassion are suggested and discussed, along with self‐management movements for the jaw and cervical region. This topic is addressed in two sessions to allow time for a detailed exploration of sleep hygiene, eating habits, and physical exercises, as well as the execution of self‐management exercises involving the cervical and orofacial regions.	Mindfulness practice focusing on breathing and body awareness, with an emphasis on the orofacial region. Sharing experiences from the previous week. Presentation on daily life habits with reflections and group discussions on eating habits. Self‐management with jaw exercises to promote mobility and lubrication: a. Carefully opening and closing the mouth while keeping the tongue tip against the palatal papilla. b. Gently lateralizing the jaw by sliding the lower teeth along a wooden spatula, ensuring not to bite down. Presentation on physical exercise routines and sleep hygiene, followed by reflections and group sharing. Self‐management with neck and shoulder exercises: a. Rotating the neck (“no” movement). b. Tilting the head up and down (“yes” movement). c. Inclining the head toward the shoulder on both sides (“maybe” movement). d. Rotating the shoulders forward and backward. Instructions for weekly practice: a. Choose a moment in the day and two exercises to repeat up to 10 times, varying exercises daily. b. Perform the movements with gentleness, openness, and curiosity, maintaining mindful attention to movement details and breath coordination.
**Week 7**	**“Connecting Emotions and Sensations"**	In this session, participants are invited to engage in a brief mindfulness practice focused on body awareness, followed by functional orofacial exercises to support self‐management of TMD with mindful attention. This involves recognizing, moment by moment, the physical sensations elicited by the exercises, as well as any thoughts about these sensations and the proposed movements. Participants may also notice emotions related to comfort or discomfort, pleasure or displeasure during the exercises. This process facilitates awareness of the transient nature of emotions and their potential association with physical sensations, which are also temporary. Recognizing the natural flow of emotions as part of one's existence provides a foundation for empathy and self‐compassion.	Mindfulness practice during cervical movements with the neck (“yes, no, maybe”) and the jaw (gentle, slow mouth opening with the tongue tip resting on the palatal papilla). Guidance for recognizing pleasant, unpleasant, or neutral physical sensations, as well as associated emotional experiences, with the intention of embracing what arises in the present moment. Brief sharing of the previous practice and weekly experiences. Review of the importance of sleep and ways to improve it: Participants are asked to reflect and provide suggestions based on guiding questions such as: Do you do anything to sleep well? What helps you sleep better? What might disrupt your sleep?
**Week 8**	**“Sharing Experiences and Extending Learning: Real‐Life Insights”**	In this session, the focus is on recognizing the challenges of integrating self‐management practices and mindfulness into daily life. Participants refine their awareness of habits and skills through mindfulness practice with an emphasis on the orofacial region. This is an opportunity to explore in greater depth the ways to integrate new habits into daily life with discipline, flexibility, acceptance, and ease, considering the dynamic nature of life and the ever‐changing circumstances. Participants are reminded that cultivating new habits and mindfulness is an ongoing process that requires empathy, compassion, and loving‐kindness, leading to greater well‐being and a sense of peace. They are also told that while this marks the final session of the program, it is the beginning of the rest of their lives.	Brief mindfulness practice focusing on breathing. Sharing reflections on the past week, discussing challenges and successes, and exploring opportunities to incorporate new self‐care habits, including exercises, mindfulness practices, and home strategies for pain relief. Repetition of mandibular and cervical exercises while practicing mindful awareness—paying attention to the experience of each movement moment by moment, with gentleness, curiosity, acceptance, and self‐compassion. Farewell.

## 4 Discussion

The findings of this study highlight participants’ perceived benefits and experiences with the Mindfulness‐Based Orofacial Pain Education Program, suggesting that mindfulness‐based education may support self‐awareness and self‐management of orofacial pain. Thematic Network Analysis [22, 23] identified that social support played a critical role in enhancing engagement, facilitating a shift from a biomedical to a biopsychosocial understanding of pain. Participants who demonstrated adherence to mindfulness practices reported improved self‐efficacy, heightened pain awareness, and a stronger capacity for self‐management. However, environmental barriers and perceived monotony in exercises emerged as challenges, potentially impacting sustained engagement.

These findings suggest that mindfulness‐based practices may facilitate adaptive emotional regulation and a more reflective relationship with pain, supporting participants’ ability to manage daily discomfort. They are consistent with prior evidence that mindfulness promotes attentional control, stress reduction, and acceptance of present‐moment experiences [13, 14, 15, 16, 18, 19, 20]. By cultivating these skills, participants appeared to shift from a reactive to a more reflective relationship with pain, allowing greater calmness and self‐compassion during symptom exacerbation [24, 25].

Social support emerged as a crucial facilitator of engagement, reinforcing adherence to mindfulness and self‐management exercises. Participants emphasized that sharing their experiences within the group helped them feel understood and supported. This aligns with the minority stress framework, which highlights the protective role of affirming social contexts for transgender individuals exposed to chronic stress and discrimination [26, 27, 28, 29, 30]. Within this supportive group environment, participants were able to express vulnerability, exchange coping strategies, and strengthen their sense of belonging, contributing to emotional resilience [31, 32].

The development of autonomy in pain management was another key outcome. Participants described applying mindfulness‐based breathing and relaxation techniques in daily life, suggesting increased self‐efficacy and internalization of learned strategies. These behavioral changes corroborate previous studies showing that mindfulness facilitates cognitive reappraisal, enhances self‐regulation, and reduces pain catastrophization [14, 18, 24, 25, 33, 34, 35, 36, 37, 38, 39, 40]. These challenges reflect contextual limitations commonly observed in mindfulness‐based interventions delivered in public clinical settings [36, 37, 38, 39]. Future adaptations could include hybrid or digital formats to ensure the flexibility and sustainability of engagement [37, 38, 39, 41].

Body dissatisfaction and gender dysphoria also play a significant role in the pain experience among transgender individuals. Research has indicated that distress related to one's physical appearance, particularly in relation to secondary sex characteristics, can contribute to heightened somatic symptoms and increased pain sensitivity [29]. This aligns with findings that transgender individuals with unresolved gender dysphoria report higher rates of musculoskeletal tension and chronic pain [27].

Participants who described greater consistency in their practice reported more noticeable improvements in self‐regulation. These experiences highlight the importance of tailoring mindfulness education to individual readiness and contextual factors. The adaptation of mindfulness‐based interventions for vulnerable populations requires careful consideration of sociodemographic factors that may influence adherence, engagement, and therapeutic outcomes. Research indicates that marginalized communities, including racial/ethnic minorities, low‐income individuals, and LGBTQ+ populations, experience higher chronic stress and reduced healthcare access, which can impact the effectiveness of mindfulness practices [30, 31]. Furthermore, exposure to discrimination and social exclusion contributes to heightened physiological stress responses, which can affect how mindfulness‐based pain management is perceived and practiced [30, 32]. These findings align with previous studies suggesting that mindfulness programs must be tailored to reduce contextual obstacles, ensuring inclusivity and accessibility for individuals facing systemic inequalities [30, 32].

Social support is known to enhance motivation and sustain participation in health‐related interventions, largely through group cohesion—defined as the development of unity, interdependence, and identification with the collective. Strong cohesion fosters an environment that encourages sharing, acceptance, and participation, reduces loneliness, and increases the sense of belonging, all of which strengthen interpersonal bonds and provide essential social support. [33, 40] In this study, the group setting promoted belonging, reduced isolation, and enhanced emotional resilience, enabling mutual learning and validation among participants. These findings align with prior research showing that group‐based mindfulness programs achieve higher retention and improved psychological outcomes [34].

Another key strength was the observed development of autonomy in pain management. Participants reported gaining valuable self‐awareness and self‐regulatory skills, which are critical for the long‐term sustainability of pain management strategies. The results support existing literature indicating that mindfulness facilitates cognitive reappraisal, enhances emotional acceptance, and reduces pain catastrophization [25]. The ability to regulate pain perception through mindfulness has been well‐documented in neurophysiological studies, which have identified the involvement of higher‐order brain regions in modulating pain‐related distress [14].

Despite these benefits, some barriers to adherence emerged, particularly environmental distractions and the repetitive nature of exercises. Some participants reported difficulty maintaining focus due to external stimuli, indicating that the intervention setting may significantly influence adherence to mindfulness practice—an observation consistent with research emphasizing the value of structured and private environments for optimizing engagement [36]. Future adaptations should consider hybrid delivery models that combine in‐person and digital formats to offer greater flexibility and allow participants to practice in more controlled settings [37]. Such flexible approaches may enhance accessibility and better accommodate the diverse needs of participants [38, 39].

Despite the strengths of this study, some limitations should be acknowledged. The qualitative design and small sample size limit the generalizability of the findings. Although fifteen individuals completed the Mindfulness‐Based Orofacial Pain Education Program, only four consented to the qualitative phase, which may not fully represent the diversity of participant experiences. The short intervention period and lack of long‐term follow‐up prevent conclusions about the durability of reported benefits. As this was the first application of the Mindfulness‐Based Orofacial Pain Education Program, no prior efficacy data are available, and future controlled and longitudinal studies should evaluate its effects on pain intensity, function, and psychosocial outcomes.

## 5 Conclusion

This study highlights participants’ perceived benefits and experiences with the Mindfulness‐Based Orofacial Pain Education Program, suggesting its potential to promote self‐awareness, emotional regulation, and self‐management of orofacial pain. The findings underscore the clinical relevance of incorporating mindfulness‐based educational strategies into orofacial pain care, as they may enhance patient engagement and support a shift toward a biopsychosocial understanding of pain. Future research should refine and test these approaches through longitudinal designs with larger samples, examining their long‐term impact, scalability, and integration into multidisciplinary pain management settings.

## Author Contributions

MOM and LVM conceived and designed the study, coordinated the multidisciplinary research team, and supervised all stages of implementation, from intervention planning to data interpretation. They were primarily responsible for drafting the manuscript, critically revising the content for intellectual coherence, and ensuring methodological rigor throughout the study process. BD, AMM, NSM, and VHARS contributed to participant recruitment, data collection, transcription of audio materials, and preliminary analysis of both qualitative and quantitative components, ensuring the reliability and consistency of the dataset. LGR conducted the qualitative analyses using Thematic Network Analysis and was responsible for coding, interpreting, and integrating the thematic findings into the broader conceptual framework of the study. LASL, CRALP, and ECSGD provided theoretical and methodological guidance, contributing to the articulation between psychological, behavioral, and nursing perspectives and the clinical aspects of orofacial pain and mindfulness education. JFMC coordinated the clinical phase of the project, supervised patient flow and eligibility screening at the TMD/OFP clinic, ensured ethical compliance, and performed a final critical review of the manuscript for scientific accuracy and clinical relevance. All authors contributed substantially to the work, reviewed and approved the final version of the manuscript, and agree to be accountable for all aspects of its content and integrity.

## Funding

The authors gratefully acknowledge financial support from Coordination for the Improvement of Higher Education Personnel (CAPES, Brazil, 33002029032P4) and São Paulo Research Foundation (FAPESP, Brazil, 2025/11104‐9, 2024/23302‐7, 2025/15283‐5).

## Ethics Statement

The study protocol was approved by the Research Ethics Committee (CAAE: 82989524.5.0000.5419), in accordance with the guidelines of Resolution No. 466/2012 of the Brazilian National Health Council.

## Supporting information




**Domain 1**: Research team and reflexivity.
**Domain 2**: Study design.
**Domain 3**: Analysis and findings.
