# Supplementary material for: Mindfulness‐Based Orofacial Pain Education and Management Program: A Qualitative Analysis of Transgender Participants’ Perceptions
Source: Spec Care Dentist. 2026 Jan 28;46(1):e70141. doi: 10.1111/scd.70141 (PMC12848843; doi:10.1111/scd.70141)
Supplement: Supplementary file 1 — Domain 1: Research team and reflexivity. Domain 2: Study design. Domain 3: Analysis and findings. [file SCD-46-0-s001.docx]

**COREQ – Consolidated Criteria for Reporting Qualitative Research**

**Study:** *Mindfulness-Based Orofacial Pain Education Program: Participants’ Perceptions and Engagement*
**Authors:** Magri LV et al.
**Journal:** *Special Care in Dentistry* (submission version)

**Domain 1: Research team and reflexivity**

| **Item** | **Description** | **Reported on page** |
| --- | --- | --- |
| 1. Interviewer/facilitator | The focus groups were conducted by an experienced researcher in psychology and orofacial pain, not involved in intervention delivery. | p. 5 |
| 2. Credentials | All authors hold advanced degrees (PhD, DDS, MSc) in dentistry, psychology, or health sciences. | p. 2 |
| 3. Occupation | The facilitators were academic researchers and clinicians specializing in TMD/orofacial pain. | p. 2 |
| 4. Gender | The lead researchers and facilitators were female. | p. 2 |
| 5. Experience and training | The research team had prior experience with mindfulness-based interventions and qualitative health research. | p. 2, 5 |
| 6. Relationship established | No prior personal relationship existed between the interviewer and participants. | p. 5 |
| 7. Participant knowledge of interviewer | Participants were informed about the interviewer’s role as a researcher studying pain education, not as a clinician. | p. 5 |
| 8. Interviewer characteristics | Reflexivity was maintained through team discussions during analysis to mitigate potential bias. | p. 6 |

**Domain 2: Study design**

| **Item** | **Description** | **Reported on page** |
| --- | --- | --- |
| 9. Methodological orientation and theory | Thematic Network Analysis (Attride-Stirling, 2001) was used, guided by an inductive, postpositivist approach. | p. 6 |
| 10. Sampling | Purposive convenience sampling of participants who completed the Mindfulness-Based Orofacial Pain Education Program. | p. 5 |
| 11. Method of approach | Participants were invited in person at the end of the intervention and via email follow-up. | p. 5 |
| 12. Sample size | Four participants consented to participate in the qualitative phase; saturation was achieved. | p. 5 |
| 13. Non-participation | Eleven participants completed the intervention but declined to join the qualitative phase. | p. 5 |
| 14. Setting of data collection | Focus group sessions were conducted in a private room at the university’s pain clinic. | p. 5 |
| 15. Presence of non-participants | Only the interviewer and participants were present during focus group discussions. | p. 5 |
| 16. Description of sample | Participants were adults diagnosed with chronic painful TMD, recruited from the clinical program; demographic details are presented in Table 1. | p. 5 |
| 17. Interview guide | A semi-structured script developed by the research team based on mindfulness and pain education literature. | p. 5 |
| 18. Repeat interviews | No repeat interviews were conducted. | p. 5 |
| 19. Audio/visual recording | Sessions were audio-recorded with participants’ consent. | p. 5 |
| 20. Field notes | Brief notes were taken during and after sessions. | p. 5 |
| 21. Duration | Each focus group session lasted approximately 60 minutes. | p. 5 |
| 22. Data saturation | Thematic saturation was monitored inductively; no new themes emerged after analysis of four participants. | p. 6 |
| 23. Transcripts returned | Transcripts were not returned to participants due to confidentiality protocols. | p. 6 |

**Domain 3: Analysis and findings**

| **Item** | **Description** | **Reported on page** |
| --- | --- | --- |
| 24. Number of data coders | Two independent researchers performed data coding and reconciled differences through discussion. | p. 6 |
| 25. Description of coding tree | Coding was organized hierarchically into basic, organizing, and global themes following Thematic Network Analysis. | p. 6 |
| 26. Derivation of themes | Themes were derived inductively from the data. | p. 6 |
| 27. Software | NVivo software (v.12) was used to support data organization and coding. | p. 6 |
| 28. Participant checking | Findings were not participant-validated due to the small sample and confidentiality. | p. 6 |
| 29. Quotations presented | Representative quotes were presented for each theme, with identifiers indicating participant type. | p. 7–8 |
| 30. Data and findings consistent | The findings were consistent across data sources (participants and facilitators), supporting triangulation. | p. 7–8 |
| 31. Clarity of major themes | Four major themes were clearly identified and visually represented in Figure 2. | p. 7 |
| 32. Clarity of minor themes | Subthemes were described in Table 2 with illustrative quotations. | p. 7 |
